# Awareness and Knowledge of Ionizing Radiation in Common Radiological Investigation and Associated Risks Among Medical and Applied Medical Sciences Students at Majmaah University

**DOI:** 10.7759/cureus.67261

**Published:** 2024-08-20

**Authors:** Meshari A Alali, Omar F Alsalem, Norah F Alsalem, Mohammed F Alsalem, Layla A Alzuwayyid, Mohammed M Alrashdi, Mohammed M Almasoudi, Abdulaziz A Alaskar, Aljoharah A Almaziad

**Affiliations:** 1 College of Medicine, Majmaah University, Al Majmaah, SAU; 2 College of Medicine, King Saud Bin Abdulaziz University for Health Sciences, Riyadh, SAU; 3 College of Medicine, King Saud University, Riyadh, SAU; 4 Radiology, Majmaah University, Al Majmaah, SAU

**Keywords:** radiological investigations, radiation risk, students, knowledge, awareness

## Abstract

Aim: This study aims to evaluate the awareness and knowledge of ionizing radiation in common radiological investigations, as well as the associated risks, among medical and applied medical sciences students at Majmaah University in Majmaah, Saudi Arabia.

Materials and methods: This cross-sectional study involved 209 participants who completed an anonymous electronic questionnaire consisting of 21 questions. Participants were categorized based on demographic characteristics and assessed for their awareness and knowledge of radiation and its associated risks. Data analysis was conducted using the Statistical Package for the Social Sciences (SPSS) software, version 24.0 (IBM Corp., Armonk, NY, USA). Qualitative data comparisons were performed using the Chi-square test, with a p-value of <0.05 considered statistically significant.

Results: A total of 209 participants completed the questionnaire, with a nearly equal distribution of males (49.8%) and females (50.2%). Most of the participants were medical students (59.3%). The overall knowledge score had a mean of 2 (SD=2) and a range of 0-7. The overall awareness score had a mean of 3 (SD=2) and a range of 0-6. Male gender and knowledge had a significant association (p=0.022). Applied medical sciences students had a significantly higher awareness level than medical students (p=0.004). There was a significant association between academic level and knowledge level (p=0.025), which was highest among third-year students.

Conclusion: Overall medical and applied medical sciences students’ knowledge and awareness of ionizing radiation dose and the health risks associated with it was reported to be low. The results highlight the need to train medical and applied medical sciences students with sufficient radiological education that enable them to adhere to safe practices in the future.

## Introduction

As medical science advanced, the use of radiation-based imaging technology for the purposing of diagnosis of different diseases, treatment, and assessing the effectiveness of therapy has been greatly enhanced [1]. The use of imaging technologies such as X-rays and CT scans involves radiations created from natural or artificial sources and can ionize. The ionizing radiations tend to damage the living tissues by causing chemical and structural changes in the cells [2].

To ensure public safety guidelines, principles based on as low as reasonably achievable (ALARA) are in effect. These principles ensure that the radiation doses that a person may get in whichever cases are as low as possible. Furthermore, ALARA also emphasizes reducing the time to a minimum and keeping the maximum distance along with protective equipment when these radiations are in use for medical purposes [3].

It is proven that if precautionary measures are not taken, the person can be severely affected by these radiations. The harmful effects of radiation on biological tissues can be categorized into deterministic and stochastic effects. Deterministic effects have a specific threshold dose, meaning that surpassing this threshold causes adverse outcomes like burns and hair loss. In contrast, stochastic effects do not have a threshold, so harmful consequences like mutations, teratogenesis, and cancer can occur at any dose [4,5].

It is important to recognize the after-effects of the ionizing radiation being used in medical science, as it greatly increases the risk of cancer development in the general population and causes teratogenic effects in pregnant women, thereby posing a great threat to public health [6,7].

Healthcare workers are also exposed to this radiation during medical procedures and they are equally at risk as the patients. Therefore, these healthcare professionals must be aware of doses of radiation exposure during radiological studies used for medical purposes [8,9]. However, the radiation dose they receive is dependent on their knowledge and awareness [10].

Various studies have been conducted to analyze the knowledge and awareness of healthcare workers regarding radiation safety in medical procedures. In a survey, a Korean researcher found that nearly 61% of pain physicians had not received any radiation safety education [11]. Similarly, a study conducted in Saudi Arabia showed that 58.6% of participants had minimal knowledge related to radiation doses and exposure during routine radiological examinations [12]. Another study from Saudi Arabia found that many medical facilities lack radiation protection accessories such as lead glass, lead aprons, and shielding materials [13].

As protection from ionizing radiation, understanding radiation dose, and determining when and to whom to use ionizing radiation are critically important for the safety of patients as well as healthcare workers, this study aims to assess the level of knowledge and awareness among medical and applied medical sciences students about radiological technologies and their effect on health at Majmaah University, Saudi Arabia, as well as to identify the gaps in knowledge and areas of improvement in radiology education at the university.

## Materials and methods

Study design

This descriptive cross-sectional institutional-based study targeted the male and female student population of the College of Medicine and Applied Medical Sciences during the 2023-2024 academic year.

Study population

The study encompassed all students within the College of Medicine and Applied Medical Sciences at Majmaah University. Inclusion criteria encompassed all medical and applied medical sciences students from Majmaah University who fully agreed to participate. Exclusion criteria included students from other universities.

Data collection

Our recommended sample size was 211, with an estimated population size of 605 (medical students, medical laboratory students, medical technology, students, nursing students, physical therapy, students, and radiology students) and a margin of error of 5% at a 95% confidence level. Raosoft online sample size calculator was used. The sample size from each specialty was calculated as 391 medical students, 60 radiology students, 94 nursing students, 20 medical laboratory students, 20 medical technology students, and 20 physical therapy students. The response rate was 49.2%.

The data was collected from November 2023 to February 2024. Data was gathered using a pre-tested, self-administered questionnaire designed to assess the knowledge and perceptions of medical and applied medical sciences students regarding radiation. The validity of the questionnaire was confirmed by a radiologist. The questionnaire contained 22 multiple-choice and true-or-false questions and was divided into three sections. The first section contained items on demographic characteristics (i.e., gender, current clinical year, specialty and age). The second section contained items that tested the respondent's knowledge about their radiological knowledge and radiation safety principles. The third section contained items that tested knowledge about effective radiation doses as well as oncological radiation risks. The questionnaire, available in both Arabic and English, was created using Google Forms and distributed to the targeted population via social media platforms WhatsApp and Telegram.

Outcomes

The primary outcome of this study is to evaluate the mean score of students' knowledge and awareness regarding ionizing radiation. Additionally to compare these scores between applied medical sciences students and medical students to determine which group has a higher level of correct answers.

Statistical analyses

Descriptive statistics were used to summarize demographic characteristics and response distributions for knowledge and awareness questions. Chi-square tests were conducted to examine associations between demographic variables and levels of knowledge and awareness. Logistic regression analyses were performed to identify factors associated with good knowledge and good awareness, with odds ratios (OR) and 95% confidence intervals (CI) reported. The significance level was set at p<0.05 for all analyses. All analyses were done using IBM SPSS v29.0.0.0 (IBM Corp., Armonk, NY, USA).

Ethical approval

Ethical considerations included obtaining informed consent from all participants and maintaining data confidentiality throughout the research process. Ethical approval to conduct this study was obtained from the Majmaah University for Research Ethics Committee (MUREC) (Ethics number MUREC-Mar.25/COM-2024/11-8).

## Results

The study included a total of 209 participants, with a nearly equal distribution of males (49.8%) and females (50.2%). The majority of the participants (44.0%) were between the ages of 18 and 20, followed by those aged 21-22 (29.2%), 23-24 (18.7%), and over 24 (8.1%). The participants were from various specialties, with medical students comprising the largest group (59.3%), followed by radiology students (18.2%), nursing students (13.4%), medical laboratory students (3.3%), physical therapy students (2.9%), and medical technology students (2.9%). The participants were distributed across different academic years, with the highest representation from the first year (25.4%), followed by the third year (21.1%), second year (19.6%), fifth year (18.7%), fourth year (10.0%), and interns (5.3%) (Table 1).

**Table 1:** Demographic Characteristics of Participants

		N	N%
Gender	Male	104	49.8%
	Female	105	50.2%
Age	18-20	92	44.0%
	21-22	61	29.2%
	23-24	39	18.7%
	>24	17	8.1%
Specialty	Medical student	124	59.3%
	Radiology student	38	18.2%
	Nursing student	28	13.4%
	Physical therapy student	6	2.9%
	Medical laboratory student	7	3.3%
	Medical technology student	6	2.9%
Academic year	1st	53	25.4%
	2nd	41	19.6%
	3rd	44	21.1%
	4th	21	10.0%
	5th	39	18.7%
	Intern	11	5.3%

The study assessed the participants' knowledge of radiation in common radiological investigations and associated risks through a series of questions, with the maximum possible score being 9. The results showed that only 27.3% of the participants correctly identified the age group most sensitive to ionizing radiation. More than half of the participants (59.3%) answered that pregnant women should avoid all types of medical imaging, while 42.1% responded that it is safe for pregnant women to have a mammography (Table 2).

**Table 2:** Proportions of Correct Answers for the Knowledge Section mSv: millisievert, PA: posterior to anterior

Question	N (%)
Which one of the following age groups is the most sensitive to ionizing radiation?	57 (27.3)
Pregnant women should avoid all types of medical imaging?	124 (59.3)
Is it safe for pregnant women to have a mammography?	88 (42.1)
Regarding the risk of cancer as long-term effect of radiation exposure, which statement of the following is true:	77 (36.8)
What is the annual whole-body dose limit for a patient?	24 (11.5)
What is the radiation dose in mSv of a "PA chest X-ray"?	32 (15.3)
If the exposure to PA chest were taken as 1 unit, how many units would a patient absorb during "MRI brain"?	32 (15.3)
If the exposure to PA chest were taken as 1 unit, how many units would a patient absorb during "Ultrasound kidneys"?	34 (16.3)
What is the risk for a patient to have an oncological complication from a single abdominal CT scan?	25 (12)
Overall knowledge, Mean(SD), Range	2(2), 0-7
Median, IQR	2, 1-3

Regarding the long-term effects of radiation exposure on cancer risk, 36.8% of the participants chose that the likelihood for cancer to occur is proportional to the radiation dose. 20 mSv as the annual whole-body dose limit for a patient was picked by only 11.5% of the participants. The overall knowledge score had a mean of 2 (SD=2) and a range of 0-7, with a median score of 2 and an interquartile range of 1-3 (Table 2).

The study also evaluated the participants' awareness of radiation protection and related topics. ‘Yes’ responses to these questions were categorized as ‘correct’ for analysis purposes. More than half of the participants (59.3%) reported having taken a radiology course or rotation, either as part of their university curriculum or as an extracurricular activity. However, only 36.8% of the participants had attended a radiation protection course. Slightly more than half of the participants (55.5%) reported having lectures regarding ionizing radiation in their studies. When asked about the types of materials used for shielding inside the nuclear medicine department, 51.7% of the participants demonstrated awareness. More than half of the participants (56.5%) were aware of personal radiation monitoring devices. The importance of dose record-keeping was recognized by 69.4% of the participants, which was the highest percentage among the awareness questions. The overall awareness score had a mean of 3 (SD=2) and a range of 0-6, with a median score of 3 and an interquartile range of 2-5 (Table 3).

**Table 3:** Proportions of Correct (yes) Answers for the Awareness Section

Question	N (%)
Have you taken radiology course/ rotation either (University curriculum or extracurriculum)?	124 (59.3)
Have you ever attended a radiation protection course?	77 (36.8)
Have you ever had lectures regarding ionizing radiation in your study?	116 (55.5)
Do you know what types of materials are used for shielding inside the nuclear medicine department?	108 (51.7)
Are you aware of any personal radiation monitoring devices?	118 (56.5)
Are you aware of the importance of dose record keeping?	145 (69.4
Overall knowledge, Mean(SD), Range	3 (2), 0-6
Median, IQR	3, 2-5

When asked about their favorite source of knowledge about radiation protection, participants equally favored medical websites (33.0%) and university courses (33.0%). Medical books and other sources were each preferred by 11.5% of the participants, while research was the least favored source, chosen by only 11.0% of the participants (Table 4).

**Table 4:** Preferred Sources of Knowledge and Confidence in Knowledge of Ionizing Radiation Dose

		N	N%
What is your favorite source of knowledge about radiation protection?	Medical books	24	11.5%
	Medical website	69	33.0%
	Researches	23	11.0%
	University courses	69	33.0%
	Others	24	11.5%
How confident are you in your knowledge of the ionizing radiation dose of common radiological investigations?	Not really confident	76	36.4%
	Moderately confident	79	37.8%
	Very confident	54	25.8%

Regarding their confidence in knowledge of the ionizing radiation dose of common radiological investigations, participants were almost evenly distributed among the three levels of confidence. The largest group (37.8%) reported being moderately confident, followed closely by those who were not really confident (36.4%). Only about a quarter of the participants (25.8%) felt very confident in their knowledge of ionizing radiation doses (Table 4).

The study investigated the association between participants' demographic characteristics and their level of knowledge about radiation in common radiological investigations and associated risks. Knowledge levels were categorized as either poor or good based on the 75th percentile. 

A significant association was found between gender and knowledge level (p=0.022). Among participants with good knowledge, 58.9% were males and only 41.1% were females. Although there was no significant association between age and knowledge level (p=0.140), the proportion of participants with good knowledge tended to increase with age, with the exception of 18-20 and those over 24 years old (Table 5).

**Table 5:** Association Between Demographic Characteristics and Knowledge Level

			Knowledge			
			Poor	Good		p-Value
		N	N%	N	N%	
Gender	Male	51	42.9%	53	58.9%	0.022
	Female	68	57.1%	37	41.1%	
Age	18-20	58	48.7%	34	37.8%	0.140
	21-22	31	26.1%	30	33.3%	
	23-24	18	15.1%	21	23.3%	
	>24	12	10.1%	5	5.6%	
Specialty	Applied medical sciences	52	43.7%	33	36.7%	0.306
	Medicine	67	56.3%	57	63.3%	
Which Academic year?	1st	34	28.6%	19	21.1%	0.025
	2nd	31	26.1%	10	11.1%	
	3rd	18	15.1%	26	28.9%	
	4th	10	8.4%	11	12.2%	
	5th	20	16.8%	19	21.1%	
	Intern	6	5.0%	5	5.6%	

The association between specialty (applied medical science or medicine) and knowledge level was not significant (p=0.306). However, a higher proportion of medical students (63.3%) had good knowledge compared to applied medical science students (36.7%) (Table 5).

A significant association was found between academic year and knowledge level (p=0.025). The proportion of participants with good knowledge was highest among third-year students (28.9%) and lowest among interns (5.6%) (Table 5).

The study also examined the association between participants' demographic characteristics and their level of awareness about radiation protection and related topics. Awareness levels were categorized as either poor or good based on the 75th percentile.

No significant association was found between gender and awareness level (p=0.845). The proportion of participants with good awareness was similar for both males (50.8%) and females (49.2%). Although there was no significant association between age and awareness level (p=0.313), the proportion of participants with good awareness was highest among those aged 21-22 (36.9%) and lowest among those over 24 years old (6.2%) (Table 6).

**Table 6:** Association Between Demographic Characteristics and Awareness Level

		Awareness				
		Poor		Good		p-Value
		Count	Layer Column N %	Count	Layer Column N %	
Gender	Male	71	49.3%	33	50.8%	0.845
	Female	73	50.7%	32	49.2%	
Age	18-20	68	47.2%	24	36.9%	0.313
	21-22	37	25.7%	24	36.9%	
	23-24	26	18.1%	13	20.0%	
	>24	13	9.0%	4	6.2%	
Specialty	Applied medical sciences	49	34.0%	36	55.4%	0.004
	Medicine	95	66.0%	29	44.6%	
Which Academic year?	1st	42	29.2%	11	16.9%	0.417
	2nd	29	20.1%	12	18.5%	
	3rd	27	18.8%	17	26.2%	
	4th	14	9.7%	7	10.8%	
	5th	24	16.7%	15	23.1%	
	Intern	8	5.6%	3	4.6%	

A significant association was found between specialty and awareness level (p=0.004). A higher proportion of applied medical sc students (55.4%) had good awareness compared to medical students (44.6%). The association between academic year and awareness level was not significant (p=0.417). However, the proportion of participants with good awareness was highest among 3rdyear students (26.2%) and lowest among interns (4.6%) (Table 6).

A logistic regression analysis was performed to identify factors associated with good knowledge about radiation in common radiological investigations and associated risks. The analysis included gender, age, specialty, and academic year as independent variables.

Gender was found to be a significant predictor of good knowledge (p=0.034). Females had lower odds of having good knowledge compared to males (adjusted odds ratio (aOR)=0.489, 95% CI: 0.252-0.948). Age, specialty, and academic year were not significantly associated with good knowledge (Table 7).

**Table 7:** Logistic Regression Analysis of Factors Associated with Good Knowledge aOR: adjusted odds ratio

Knowledge, Goo			d		
	B	Sig.	aOR	95% C.I.for aOR	
				Lower	Upper
Gender, Female	-.716	.034	.489	.252	.948
Age	18-20 years, Reference category				
21-22	.210	.608	1.234	.552	2.759
23-24	.191	.750	1.210	.375	3.907
>24	-.861	.240	.423	.100	1.780
Speciality, Medical	.033	.925	1.033	.522	2.043
Academic year	1^st^ Year, Reference category				
2nd	-.609	.222	.544	.205	1.446
3rd	.824	.081	2.279	.904	5.744
4th	.525	.406	1.691	.490	5.831
5th	.273	.664	1.314	.383	4.512
Intern	.648	.439	1.911	.370	9.872

A logistic regression analysis was conducted to identify factors associated with good awareness of radiation protection and related topics. The analysis included gender, age, specialty, and academic year as independent variables. Specialty was found to be a significant predictor of good awareness (p=0.002). Medical students had lower odds of having good awareness compared to applied medical science students (aOR=0.305, 95% CI: 0.145-0.642). Gender, age, and academic year were not significantly associated with good awareness (Table 8).

**Table 8:** Logistic Regression Analysis of Factors Associated with Good Awareness aOR: adjusted odds ratio

	Awareness, Good				
	B	Sig.	aOR	95% C.I.for aOR	
				Lower	Upper
Gender, Female	-.386	.292	.680	.331	1.394
Age	18-20 years, Reference category				
21-22	.419	.321	1.520	.665	3.476
23-24	.251	.683	1.285	.385	4.291
>24	-.219	.776	.803	.178	3.628
Speciality, Medical	-1.189	.002	.305	.145	.642
Academic year	1^st^ Year, Reference category				
2nd	.083	.875	1.086	.386	3.057
3rd	.566	.268	1.761	.647	4.788
4th	.271	.690	1.311	.346	4.968
5th	.912	.166	2.490	.684	9.067
Intern	.430	.642	1.538	.251	9.430

## Discussion

In our cross-sectional study, we aimed to assess the knowledge and awareness of medical and applied medical sciences students. The key findings of our study are that it demonstrated a low level of knowledge. The mean score was 2 (SD=2) out of a possible 9, and low level of awareness with a mean score of 3 (SD=2) out of a possible 6. These results favor our hypothesis that medical and applied medical sciences students are not fully aware of radiation doses and their effects on health. These findings are in line with previous studies that demonstrated low knowledge levels of ionizing radiation [14-16]. However, a Nepalese study conducted on radiology professionals and students found that the average radiation level of awareness was 9.6 out of a maximum of 13 [17].

When we analyzed the knowledge level, only 11.5% answered correctly to the annual whole-body dose limit. This is alarming as not knowing the annual whole-body dose limit might put the person being exposed to radiation at increased risk of cancer [18]. A similar finding was seen in a Norwegian study in which only 12% correctly answered this question [15]. Another concerning finding is that only 12% knew what the risk was for a patient to have an oncological complication from a single abdominal CT scan. For an adult, the lifetime cancer risk following an abdominal CT is generally estimated to be one in 2000 [19]. This is consistent with the previous study, in which 12% answered this question correctly [15]. One of the most common and important imaging modalities performed is X-ray [20]. Unfortunately, only 15.3% responded correctly to the radiation dose of a posterior to anterior (PA) chest X-ray. A low percentage of an Australian study of 38.3% had only answered this question [21].

When we assessed for radiology-related education, our study showed that 59.3% of respondents had taken a radiology course, 36.8% had taken a radiology protection course, and 55.5% had attended a lecture on ionizing radiation, indicating a notable number of students being exposed to education related to radiation safety and protection. This was much greater than a previous study that was conducted in Saudi Arabia, where 73% of participants reported receiving no education about radiation protection [1]. 

Furthermore, in our study, similar to Najjar et al. (2022), there was a significant association between gender and knowledge level (p=0.022), and being male was a significant predictor of good knowledge (p=0.034) [1]. The association between specialty and knowledge level was not significant (p=0.306). However, students (63.3%) had good knowledge compared to applied medicine students (36.7%). A significant association was found between academic year and knowledge level (p=0.025). The proportion of participants with good knowledge was highest among third-year students (28.9%). Applied medical sciences students were significantly associated with having good awareness compared to medical students (p=0.004), and their specialty was found to be a significant predictor of good awareness (p=0.002).

The low level of knowledge and awareness among medical and applied medical sciences students regarding radiation doses and their potential health effects emphasizes the need for enhanced education for present and future doctors. Given that a significant proportion (59.3%) of students have been exposed to radiology courses and rotations yet still demonstrate inadequate understanding, it is clear that the current curriculum taught at the university may be insufficient. To address this problem, it is crucial to implement more effective educational strategies, such as radiation safety courses, into the curriculum and offer regular, targeted training sessions. These efforts are essential to ensuring that future healthcare practitioners are well-educated to minimize radiation risks to their patients and themselves. 

This research has a few limitations. The reliance on self-reported data could also introduce response bias, as participants may overestimate or underestimate their knowledge and practices. This study is limited specifically to the students of only Majmanah University, which reduces the generalizability of the results. The study revealed significant gaps in knowledge and awareness about radiation in common radiological investigations among medical and applied medical sciences students at Majmaah University.

## Conclusions

Participants demonstrated low levels of knowledge and awareness. Male gender was found to be a significant predictor of knowledge, and applied medical specialty was a significant predictor of awareness. In order to increase the level of knowledge and awareness of radiation, more radiation courses should be added to the curriculum. Knowledge and awareness may also differ in the region where the study is conducted; therefore, it is recommended that future research include a multi-centric study.

## Appendices

**Table 9:** Section 1

Sex:	Male
	Female
	I prefer not to answer
Age:	18-20
	21-22
	23-24
	24<
Specialty:	Medical student
	Radiology student
	Nursing student
	Physical therapy student
	Medical laboratory student
	Medical technology student
Which Year:	1st
	2nd
	3rd
	4th
	5th
	6th
	Intern

**Table 10:** Section 2

Have you taken radiology course/rotation either (University curriculum or extra curriculum)?	No
	Yes
Have you ever attended a radiation protection course?	Yes, I have
	No, I haven’t
Have you ever had lectures regarding ionizing radiation in your study?	Yes
	No
	I don’t know
Which one of the following age groups is the most sensitive to ionizing radiation?	Adolescent
	Adult
	Children
	Elderly
	Embryo
	Pregnant women
	I do not know
Pregnant women should avoid all types of medical imaging:	True
	False
	I do not know
Is it safe for pregnant women to do a mammography?	Yes
	No
	I do not know
Regarding the risk of cancer as long-term effect of radiation exposure, which statement of the following is true:	Cancer will not always occur, but its likelihood is proportional to the radiation dose
	Cancer will not always occur, and its likelihood is not proportional to the radiation dose
	Cancer risk is one of the deterministic effects of radiation exposure
	Cancer after radiation exposure occurs in children only
	I do not know
Do you know what types of materials are used for shielding inside the nuclear medicine department?	Yes
	No
Are you aware of any personal radiation monitoring devices?	Yes
	No
Are you aware of the importance of dose record keeping?	Yes
	No
What is your favorite source of knowledge about radiation protection?	Medical website
	University courses
	Researches
	Medical books
	Others
How confident are you in your knowledge of the ionizing radiation dose of common radiological investigations?	Moderately confident
	Not really confident
	Very confident

**Table 11:** Section 3

What is the annual whole-body dose limit for a patient?	mSv 5
	20 mSv
	50 mSv
	100 mSv
	200 mSv
	2 Gy
What is the radiation dose in mSv of a "PA chest X-ray"?	0.02
	0.2
	20
	I Don't know
If the exposure to PA chest were taken as 1 unit, how many units would a patient absorb during "MRI brain"?	0-20
	21-80
	80-200
	201-500
	>500
	I Do not know
If the exposure to PA chest were taken as 1 unit, how many units would a patient absorb during "Ultrasound kidneys"?	0-20
	21-80
	80-200
	201-500
	>500
	I Don't know
What is the risk of inducing a fatal cancer to a patient from a single CT scan of the abdomen?	1 in 200
	1 in 2000
	1 in 20,000
	1 in 200,000
